# Molecular detection of *Paragonimus mexicanus* in freshwater crabs: new records in Oaxaca, Mexico

**DOI:** 10.1590/S1984-29612026019

**Published:** 2026-07-06

**Authors:** Jaime Vargas-Arzola, Ericel Hernández-García, Juan Pablo Merino-Villalobos, Mario Alfredo Urbina-Mata, Aristeo Segura-Salvador, Nancy Gabriela Santos-Hernández, Christian Ruiz Castillejos, Dolores Guadalupe Vidal-López, Emilio Ismael Romero-Berny, Adriana Moreno-Rodríguez, José Antonio De Fuentes-Vicente

**Affiliations:** 1 Universidad Autónoma Benito Juárez de Oaxaca – UABJO, Facultad de Ciencias Químicas – FCQ, Oaxaca, OAX, México; 2 Universidad de Ciencias y Artes de Chiapas – UNICACH, Instituto de Ciencias Biológicas – ICBIOL, Laboratorio de Investigación y Diagnóstico Molecular, Tuxtla Gutiérrez, CHP, México

**Keywords:** Freshwater decapods, intermediate hosts, lung fluke, molecular diagnosis, Neotropical region, trematode infection, Decápodes de água doce, hospedeiros intermediários, duela pulmonar, diagnóstico molecular, região Neotropical, infecção por trematódeos

## Abstract

Paragonimiasis is a zoonotic disease caused by lung flukes of the genus *Paragonimus*, which use freshwater snails and crustaceans as intermediate hosts and mammals as definitive hosts. Despite its public health importance, few studies have documented the distribution of this parasite in tropical regions of Mexico. In April 2024, a total of 180 freshwater crabs of the genus *Pseudothelphusa* were collected from five localities in Oaxaca State, Mexico. Metacercariae were observed in 47.2% of the crabs examined, with the highest mean intensity (18.46 cysts per crab) recorded in San Pedro Molinos locality. Morphologically, the metacercariae corresponded to *Paragonimus mexicanus*, although a distinct pink morphotype resembling *P. caliensis* was detected in San Juan Cacahuatepec locality. Molecular identification based on the 28S rRNA gene confirmed a 98.51% to 98.72% similarity with *P. mexicanus* (GenBank accession no. HM172619.1). This study provides new molecular records of *P. mexicanus* in freshwater crabs from previously unsampled localities in Oaxaca, expanding the known geographic distribution of the parasite in southern Mexico. Continuous surveillance and molecular characterization of *Paragonimus* species are essential to clarify their diversity and assess zoonotic risk in Neotropical freshwater systems.

## Resumo

A paragonimíase é uma zoonose causada por trematódeos do gênero *Paragonimus*, que utilizam caramujos e crustáceos de água doce como hospedeiros intermediários e mamíferos como hospedeiros definitivos. Apesar de sua importância em Saúde Pública, poucos estudos documentaram a distribuição desse parasito em regiões tropicais do México. Em abril de 2024, foram coletados 180 caranguejos de água doce do gênero *Pseudothelphusa* em cinco localidades do estado de Oaxaca, México. Metacercárias foram observadas em 47,2% dos caranguejos examinados, com a maior intensidade média (18,46 cistos por caranguejo) registrada na localidade de San Pedro Molinos. Morfologicamente, as metacercárias corresponderam a *Paragonimus mexicanus*, embora um morfotipo rosado, semelhante a *P. caliensis*, tenha sido detectado na localidade de San Juan Cacahuatepec. A identificação molecular, baseada no gene 28S rRNA, revelou 98.51% - 98,72% de similaridade com *P. mexicanus* (GenBank accession no. HM172619.1). Este estudo fornece novos registros moleculares de *P. mexicanus* em caranguejos de água doce provenientes de localidades previamente não amostradas em Oaxaca, ampliando a distribuição geográfica conhecida do parasito no sul do México. A vigilância contínua e a caracterização molecular das espécies de *Paragonimus* são essenciais para esclarecer sua diversidade e avaliar o risco zoonótico em ecossistemas neotropicais de água doce.

Paragonimiasis is a food-borne parasitic disease caused by trematodes of the genus *Paragonimus*, commonly known as lung flukes. This genus comprises nearly 50 species that mainly use freshwater crustaceans as second intermediate hosts and a broad range of mammals as definitive hosts, including carnivores such as canids, felids, procyonids, and mustelids, and occasionally suids, rodents, and primates, including humans. Adult flukes inhabit the lungs of definitive hosts, particularly carnivores, where infections may cause chronic cough, dyspnea, loss of body condition, and, in severe cases, focal pneumonia, pulmonary hemorrhage, or pleural adhesions (Gallardo et al., 2014; Dubey, 2023). In other mammals, infections are often subclinical or detected *post mortem* (Blair et al., 1999; Nawa et al., 2014). In humans, pulmonary paragonimiasis may mimic tuberculosis due to overlapping respiratory symptoms (Song et al., 2014). Although the global prevalence remains uncertain, it is estimated that about 23 million people are infected and nearly 300 million are at risk, particularly in tropical and subtropical regions where suitable hosts are present (Rosa et al., 2020).

Freshwater crustaceans, particularly brachyuran crabs of the family Pseudothelphusidae, play a crucial role in the *Paragonimus* life cycle as second intermediate hosts. After developing within freshwater snails, cercariae penetrate crabs and encyst as metacercariae in their muscles and viscera, which become infective to mammals. The distribution of Paragonimiasis is therefore strongly linked to the ecology and local consumption of pseudothelphusid crabs, which are commonly harvested and eaten in several Neotropical regions of America (Vargas-Arzola et al., 2014; Bonilla-Liberato, 2025). In addition to human consumption, these crustaceans also represent a natural infection route for wild and domestic carnivores that prey upon or scavenge infected crabs, maintaining the parasite’s life cycle in rural and sylvatic environments. Consequently, pseudothelphusid crabs act as a key bridge between aquatic and terrestrial ecosystems, sustaining transmission among wildlife, domestic animals, and humans in endemic areas.

Knowledge of the geographic distribution and host range of *Paragonimus* is fundamental for zoonotic disease management, as it supports the design of preventive and control strategies at the human–animal–environment interface. However, molecular diagnosis of *Paragonimus* species in animal hosts is still rarely performed in most Neotropical countries, despite its high value for accurate species identification and confirmation of transmission cycles. Therefore, documenting the occurrence and prevalence of the parasite in new regions through molecular techniques provides robust and reliable evidence for tracing infection sources and assessing potential transmission zones (Altun et al., 2023).

**Figure 1:**
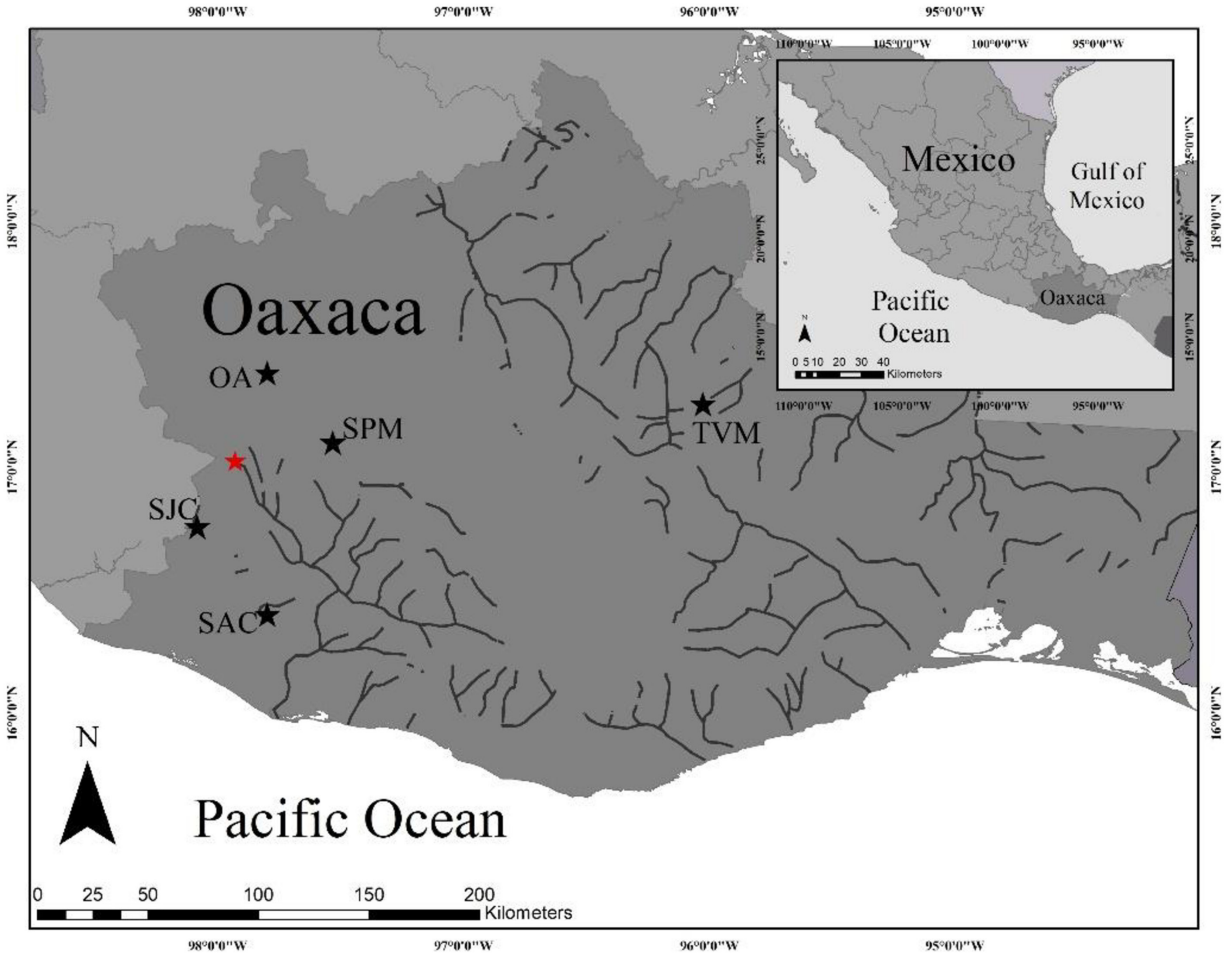
Map of Oaxaca State, Mexico, showing sampled localities with records of *Paragonimus mexicanus* in *Pseudothelphusa* crabs (black stars). The previous record of *P. mexicanus* in *Tehuana guerreroensis* (red star) is also indicated. Locality Ids: OA = Ojo de Agua, SAC = San Agustín Chayuco, SJC = San Juan Cacahuatepec, SPM = San Pedro Molinos, TVM = Totontepec Villa de Morelos.

In April 2024, a total of 180 freshwater brachyuran crabs of the genus *Pseudothelphusa* were collected from rivers and streams across five localities in three regions of Oaxaca State, Mexico (Figure 1 and Table 1), covering an altitudinal range from 259 to 2,137 m a.s.l. Specimens were collected by manual sampling in shallow sections of rivers and streams, using hand capture under rocks and submerged substrates, and sampling was conducted opportunistically across accessible sites within each locality. All individuals were examined to confirm their taxonomic identity (Villalobos-Hiriart, 2005), although specimens were identified only to the genus level. Both crabs and parasites were preserved in 70% ethanol and deposited in the Crustacean Collection of the Facultad de Ciencias Químicas (UABJO) for future reference.

**Table 1:** Sampled localities in Oaxaca, Mexico. T° = water temperature, N = number of crabs collected.

**ID**	**Locality**	**Municipality/Region**	**Latitude N**	**Longitude W**	**Altitude (m.a.s.l.)**	**T°**	**pH**	**N**
OA	Ojo de Agua	H. Tlaxiaco/Mixteca	17°23’	97°64’	2137	26.1-29.7	7.0-7.6	50
SAC	San Agustín Chayuco	Santiago Jamiltepec/Costa	16°24’	97°48’	259	22.3-23.6	7.5-7.9	50
SJC	San Juan Cacahuatepec	Cacahuatepec/Costa	16°46’44’’	98°09’12’’	442	24.3-24.9	7.1-7.8	30
SPM	San Pedro Molinos	H. Tlaxiaco/Mixteca	17°06’	97°32’	2071	23.3-23.6	7.5-7.9	30
TVM	Totontepec Villa de Morelos	Totontepec Villa de Morelos/Mixe	17°15’24’’	96°01’37’’	1843	23.1-23.7	7.2-7.6	20

Only male specimens of different sizes were collected and maintained in plastic containers with water, sand, rocks, and plant debris from each sampling site, and transported alive to the laboratory. This approach ensured consistent taxonomic identification, as diagnostic morphological characters in pseudothelphusid crabs are primarily based on male gonopod structures. The gills, digestive tract, body cavity, and leg and chela muscle tissues were examined under a stereomicroscope to detect metacercariae. When present, metacercariae exhibiting the typical ovoid morphology of lung flukes (*Paragonimus* spp.) were carefully isolated. Preliminary identification was based on general morphological features and tissue localization, and all specimens were subsequently subjected to molecular confirmation.

Since the aim of the study was the molecular detection of *Paragonimus* spp., metacercariae from each locality were pooled into a single composite sample per site prior to DNA extraction to obtain sufficient genetic material for amplification. Total genomic DNA was extracted using the Quick-DNA™ Microprep Plus Kit (Zymo Research), yielding a final concentration of 60 ng/µl (purity ratios: A260/280 = 1.6; A260/230 = 1.5). A fragment of the nuclear ribosomal 28S rRNA gene was amplified using the primers 28S-F (5′-GAGGGTGAAAGGCCCGTGGG-3′) and 28S-R (5′-ACGCATGCACACACCTGCRAGCCG-3′) (Vargas-Arzola et al., 2014). PCR amplification was performed in a total volume of 25 μl containing 100 ng of DNA template, 0.8 μM of each primer, and Master Mix (Roche). Thermal cycling conditions consisted of an initial denaturation at 92 °C for 5 min; followed by 30 cycles of 92 °C for 30 s, 61 °C for 30 s, and 72 °C for 1 min; and a final extension at 72 °C for 4 min. Negative controls were included in the PCR assays. Amplicons were visualized on 1.5% agarose gels, and PCR products were sequenced bidirectionally at the Instituto de Biotecnología, Universidad Nacional Autónoma de México. Consensus sequences were aligned using the MUSCLE algorithm (Edgar, 2004) implemented in MEGA X software (Kumar et al., 2018), with minor manual corrections. The resulting sequences were compared with reference data using BLAST searches on the NCBI platform.

A high prevalence of metacercariae was detected in all five sampled localities (Table 2). Of the 180 crabs examined, 85 specimens (47.2%) were positive for *Paragonimus* spp. metacercariae. The proportion of infected crabs differed significantly among localities (chi-square test of independence, χ^2^ = 85.77, p < 0.001). Differences in infection probability among localities were further assessed using a binomial generalized linear model (GLM), followed by Tukey post-hoc comparisons, which revealed two distinct groups: high-prevalence localities (OA, SPM) and low-prevalence localities (SAC, SJC, TVM). The highest parasite load was recorded in San Pedro Molinos, where a total of 480 metacercariae were recovered, with a mean intensity of 18.46 metacercariae per crab. Metacercariae collected from different crab tissues exhibited morphological features consistent with *Paragonimus mexicanus* Miyazaki & Ishii, 1968, including the absence of a thick cyst wall and a yellowish body. In San Juan Cacahuatepec, a morphotype with a pinkish body, similar to *P. caliensis*, was observed.

**Table 2:** Prevalence of metacercariae per sampled locality. N = number of examined crabs. Letters (a,b) refers to statistical differences between localities (p value < 0.05) revealed by a binomial generalized linear model (GLM).

**Locality ID**	**N**	**N Infected crabs (%)**	**N Isolated metacercariae**
OA	50	42 (84.0)**^a^**	340
SAC	50	6 (12.0)**^b^**	10
SJC	30	4 (13.3)**^b^**	7
SPM	30	26 (86.6)**^a^**	480
TVM	20	7 (35.0)**^b^**	8

**Figure 2:**
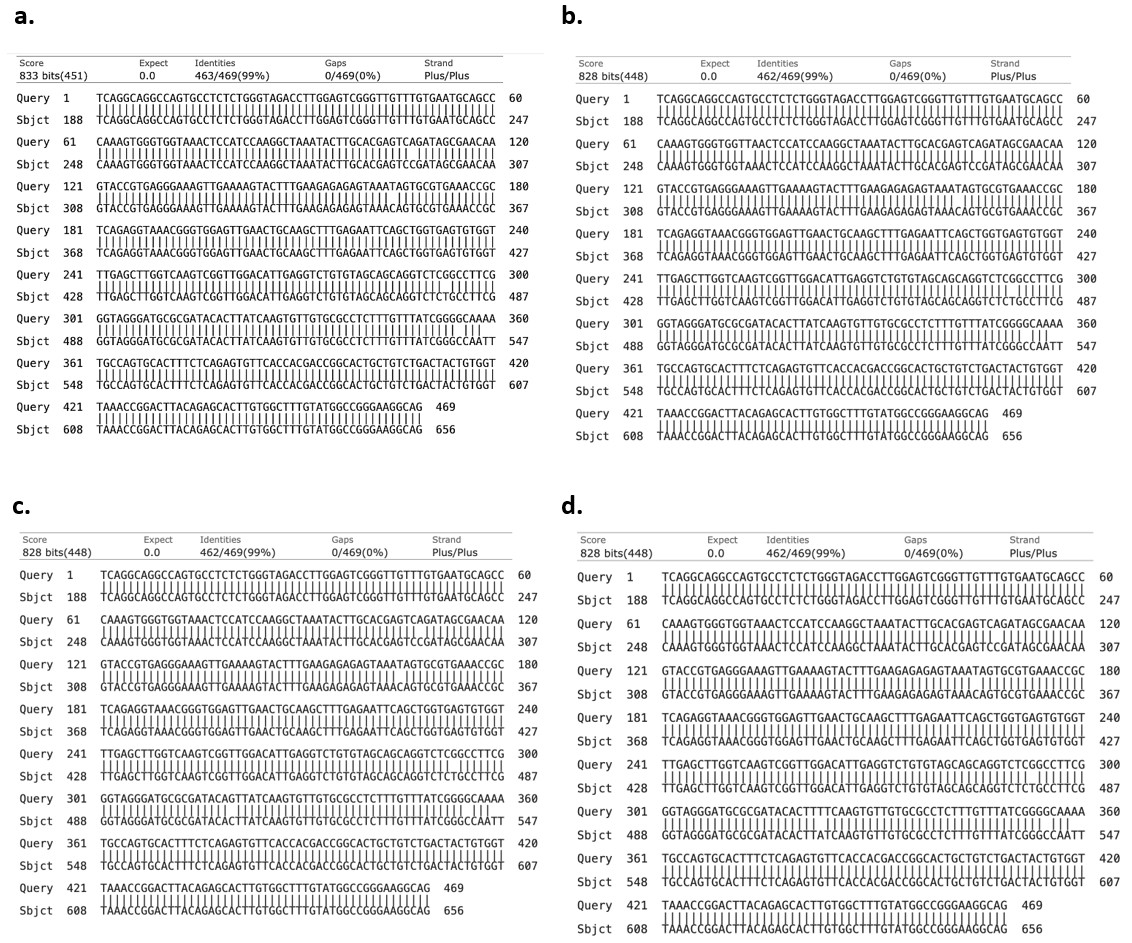
Alignment of the sample sequences (a: OA and SJC, b: SPM, c: TVM, d: SAC) corresponding to the 28S rRNA amplicon. The nucleotide sequence was analyzed in the NCBI web site. A 98.51% to 98.72% identity match was obtained between the sample sequences and *Paragonimus mexicanus* (Subject) 28S ribosomal RNA gene, partial sequence reported in GenBank (HM172619.1).

Four haplotypes were identified among the five composite samples analyzed, with a single polymorphic site detected among them. BLAST analysis of the amplified 28S rRNA gene fragment revealed a sequence identity ranging from 98.51% to 98.72% with *P. mexicanus* (GenBank accession no. HM172619.1), including the San Juan Cacahuatepec morphotype (Figure 2). This high level of similarity supports the assignment of the specimens to *P. mexicanus*. However, because the 28S rRNA gene is a relatively conserved marker (Devi et al., 2010), its discriminatory power among closely related species is limited. Despite this limitation, previous studies have demonstrated congruence between 28S-based identifications and more variable markers such as cytochrome c oxidase subunit 1 (cox1) and NADH dehydrogenase subunit 1 (nad1) in *Paragonimus* spp., supporting its usefulness for preliminary molecular identification (Devi et al., 2010).

The high infection prevalence of *P. mexicanus* in pseudothelphusid crabs from the sampled localities suggests active local transmission and a potential risk of paragonimiasis in the region. Although members of the genus *Pseudothelphusa* have previously been reported as intermediate hosts of *Paragonimus* in Mexico (Lamothe-Argumedo, 1995; Vargas-Arzola et al., 2014), the present study provides new molecularly confirmed records from localities where no previous records of *Paragonimus* infection had been reported. Notably, the high mean intensity observed in San Pedro Molinos (18.46 metacercariae per crab) may reflect favorable ecological conditions for parasite development, such as abundant intermediate snail hosts and high crab densities. In comparison, Vargas-Arzola et al. (2014) reported a 20.8% prevalence of *P. mexicanus* in *Tehuana guerreroensis* from Oaxaca, with an average of only 1.9 metacercariae per crab, indicating substantial geographic variation in infection intensity among freshwater crab hosts.

Ecological factors such as the broad ecological tolerance of the parasite, the widespread presence of suitable snail intermediate hosts, and favorable local environmental conditions likely contribute to the persistence of transmission in the region. Although the high 28S sequence similarity indicates that all metacercariae correspond to *P. mexicanus*, cryptic diversity within the genus *Paragonimus* has been documented (López-Caballero et al., 2013). Therefore, complementary analyses of mitochondrial and additional nuclear markers are recommended to clarify possible intraspecific variation and refine phylogenetic relationships among Neotropical *Paragonimus* species.

In Mexico, *P. mexicanus* metacercariae have been reported in at least seven species of pseudothelphusid crabs distributed across four genera: *Pseudothelphusa* (four species), *Odontothelphusa* (one species), *Raddaus* (one species), and *Tehuana* (one species). For the state of Oaxaca, the only previous molecularly confirmed record corresponds to *P. mexicanus* infecting *T. guerreroensis* (Vargas-Arzola et al., 2014), a species currently regarded as *Pseudothelphusa guerreroensis* following recent taxonomic revisions (Moreno-Juárez et al., 2022) and therefore reported in the literature under both generic names. The present study provides additional molecular evidence of *P. mexicanus* in pseudothelphusid crabs from Oaxaca, expanding the current knowledge of its distribution in the region. The detection of a distinct morphotype in San Juan Cacahuatepec suggests the possible coexistence of additional *Paragonimus* lineages in southern Mexico. However, because metacercariae were pooled per locality to obtain sufficient DNA for amplification, the present approach does not allow assessment of genetic variation within localities. Therefore, the results should be interpreted as representative of locality-level detection rather than individual-level diversity.

Comparable molecular analyses of *P. mexicanus* from South America and Mexico suggest relative genetic stability within ribosomal regions, indicating a broadly conserved lineage across Neotropical areas. In Ecuador, Calvopina et al. (2018) confirmed *P. mexicanus* through sequencing of the ITS2 region of metacercariae obtained from the freshwater crab *Hypolobocera guayaquilensis* in Manabí Province, showing high sequence similarity with reference sequences from GenBank. Similarly, *P. mexicanus* infecting *T. guerreroensis* from Oaxaca exhibited comparable sequence similarity and phylogenetic placement within the *P. mexicanus* clade (Vargas-Arzola et al., 2014). These findings suggest limited genetic divergence among *P. mexicanus* populations throughout its known range, although local variation in metacercarial morphology may indicate the presence of unrecognized intraspecific lineages or host-associated adaptations.

Together, these results highlight the importance of continued molecular surveillance of *Paragonimus* in freshwater crabs and potential definitive hosts in southern Mexico. Given the parasite’s zoonotic potential, increased awareness among rural communities and veterinary practitioners regarding transmission risks is essential to prevent infections in both animals and humans. Further research is needed to clarify the diversity and phylogeography of *Paragonimus* species in the Neotropics, as well as their ecological interactions with freshwater crab hosts and mammalian reservoirs. The identification of crabs at the genus level was considered sufficient for the objectives of this study, which focused on detecting the presence of *Paragonimus*. However, future investigations should incorporate species-level identification to assess host specificity and environmental determinants, thereby improving the understanding of transmission dynamics of this zoonotic trematode.
